# A Case of Secondary Fœtus

**Published:** 1854-02

**Authors:** B. A. Jesse

**Affiliations:** Shelby Co., Ky.


### Art. IV.—A CASE OF SECONDARY FŒTUS.

A LETTER TO PROF. MILLER, BY B. A. JESSE, M. D., OF SHELBY CO., KY.

Jesse's Store, *Shelby county Ky.*, Jan. 14th, 1854.

Professor Miller.

*Dear Sir:*—A circumstance so strange and unaccountable happened a few days since, in my obstetrical practice, that I wish to communicate the facts to you, for a solution of the mystery. I will go somewhat into detail, that you may have the entire history of the case.

A mulatto woman, a slave, about twenty-five years old, healthy, the mother of several children, was delivered on the 14th of April, 1853, of a healthy looking, male child, which died in a few days, of convulsions. She was delivered again the 11th day of the present month, of a still-born child of good size, with short and deformed legs and arms. Umbilical cord short, with an aneurismal sac about one or two inches from the umbilicus. The waters excessive in quantity, so much so, that she complained, for some time before her confinement, of pain and uneasiness from the distention. The labor was not protracted nor severe. The placenta delivered; and all seemed to be going on well. On the second day after her delivery I was sent for, to see her, "as there was something wrong." I went to see her; she said that the night before, "something came away," and it was attached to a cord or string, and she had tried to pull it away but could not. I examined her, and found suspended to the umbilical cord a fœtus of about six weeks or two months old. I traced the cord to the mouth of the uterus, but owing to the contracted state of the os uteri I did not attempt to insert my hand. I used very moderate traction on the cord and it parted from its attachment. There must have been another placenta, for I am satisfied that I extracted all that belonged to the first child.

Counting four weeks from the birth of her child in April, 1853, it would be about eight months to her last confinement; but I suppose any one judging merely from the size and appearance of the child would have believed she had gone full nine months. Can it be possible that conception took place in the sixth or seventh month of the first child? Was it a dwarf? Hardly, for it was not developed, not matured in its members, but just as we find a fœtus of six weeks or two months. I should be pleased to know whether or not you have met with any case similar; will you please favor me with your views in regard to it. I will add that the woman never had *twins* before, though I have delivered her of two children before this, similarly deformed.

Very Respectfully,

B. A. JESSE.

---

### PROFESSOR MILLER'S REPLY.

Louisville, Jan. 20, 1854.

Dr. B. A. Jessee,

*Dear Sir:*—Your letter of the 14th inst., was duly received, and I thank you for the narrative it gives of a rare though not unique, case in obstetric practice. It has never happened to me to meet with such a case in my own clinical experience, but several such may be found on record. Not to refer to other authors, Dr. John Ramsbotham relates a few cases of the kind in his "*Practical Observations in Midwifery*," part ii., prefacing them by some explanatory remarks. He denominates, not very happily, as I think, the abortive product a *secondary fœtus*, and considers it to be twin of the living child, or the one that attains its maturity. In the cases related by him, there were symptoms of abortion at the period of pregnancy, when this is most liable to occur, but these symptoms subsided without the ovum's being expelled, and hence he inferred that the threatening of abortion was consequent to the loss of vitality on the part of one of the ova of a twin conception. The dead ovum may be retained in the cavity of the uterus till the living one acquires its complete development, and be expelled subsequently to it at the time of parturition, as it happened in your case. You do not state whether your patient was threatened with abortion in the course of her pregnancy, and it would be interesting to make inquiries on this point.

That one of the ova in cases of twin pregnancy may be blighted without implicating the other, is proved by the observation that the dead ovum may be expelled, whilst the living one is retained, its development proceeding to maturity without molestation. The author, whom I have cited, gives cases of this kind also, in his valuable repository of facts, to which reference has been made.

If this explanation be not adopted, the only hypothesis which can be assumed to account for the phenomenon, is that of superfœtation, or a second and separate impregnation. I know of no sufficient evidence to authorize a belief in superfœtation, except perhaps its occasional occurrence in females with double uterus, or possibly in females with single uterus at a very early period, say a few hours, after the first impregnation. The changes that take place in the uterus shortly after conception and the establishment of a new process, that of gestation, are, as it appears to me, quite incompatible with a superimpregnation. I hold it to be altogether impossible at so advanced a period of pregnancy as the sixth or seventh month.

To my mind the most wonderful feature of the case reported by you, is the *superfœcundity* of the woman, whose uterine apartment was to let immediately, as it seems, after its evacuation on the 14th April, 1853, and was actually leased to him with the "short" limbs and his short-lived twin brother.

Respectfully, your obedient Serv'nt.

H. MILLER.

